# Exploring alveolar recruitability using positive end-expiratory pressure in mice overexpressing TGF-β1: a structure–function analysis

**DOI:** 10.1038/s41598-024-58213-5

**Published:** 2024-04-06

**Authors:** Franziska Roeder, Tina Röpke, Lara-Kristin Steinmetz, Martin Kolb, Ulrich A. Maus, Bradford J. Smith, Lars Knudsen

**Affiliations:** 1 Institute of Functional and Applied Anatomy, Hannover Medical School, Hannover, Germany; 2 Department of Experimental Pneumology, Hannover Medical School, Hannover, Germany; 3 Department of Medicine, Firestone Institute for Respiratory Health, McMaster University, Hamilton, ON Canada; 4 Biomedical Research in Endstage and Obstructive Lung Disease Hannover (BREATH), Member of the German Center for Lung Disease (DZL), Hannover, Germany; 5 Department of Bioengineering, College of Engineering Design and Computing, University of Colorado Denver|Anschutz Medical Campus, Aurora, CO USA; 6 Department of Pediatric Pulmonary and Sleep Medicine, School of Medicine, University of Colorado Anschutz Medical Campus, Aurora, CO USA

**Keywords:** Acute respiratory distress syndrome, Ventilation induced lung injury, TGF-β1, Recruitment, Atelectasis, Stereology, Respiration, Preclinical research, Respiratory tract diseases

## Abstract

Pre-injured lungs are prone to injury progression in response to mechanical ventilation. Heterogeneous ventilation due to (micro)atelectases imparts injurious strains on open alveoli (known as volutrauma). Hence, recruitment of (micro)atelectases by positive end-expiratory pressure (PEEP) is necessary to interrupt this vicious circle of injury but needs to be balanced against acinar overdistension. In this study, the lung-protective potential of alveolar recruitment was investigated and balanced against overdistension in pre-injured lungs. Mice, treated with empty vector (AdCl) or adenoviral active TGF-β1 (AdTGF-β1) were subjected to lung mechanical measurements during descending PEEP ventilation from 12 to 0 cmH_2_O. At each PEEP level, recruitability tests consisting of two recruitment maneuvers followed by repetitive forced oscillation perturbations to determine tissue elastance (H) and damping (G) were performed. Finally, lungs were fixed by vascular perfusion at end-expiratory airway opening pressures (Pao) of 20, 10, 5 and 2 cmH_2_O after a recruitment maneuver, and processed for design-based stereology to quantify derecruitment and distension. H and G were significantly elevated in AdTGF-β1 compared to AdCl across PEEP levels. H was minimized at PEEP = 5–8 cmH_2_O and increased at lower and higher PEEP in both groups. These findings correlated with increasing septal wall folding (= derecruitment) and reduced density of alveolar number and surface area (= distension), respectively. In AdTGF-β1 exposed mice, 27% of alveoli remained derecruited at Pao = 20 cmH_2_O. A further decrease in Pao down to 2 cmH_2_O showed derecruitment of an additional 1.1 million alveoli (48%), which was linked with an increase in alveolar size heterogeneity at Pao = 2–5 cmH_2_O. In AdCl, decreased Pao resulted in septal folding with virtually no alveolar collapse. In essence, in healthy mice alveoli do not derecruit at low PEEP ventilation. The potential of alveolar recruitability in AdTGF-β1 exposed mice is high. H is optimized at PEEP 5–8 cmH_2_O. Lower PEEP folds and larger PEEP stretches septa which results in higher H and is more pronounced in AdTGF-β1 than in AdCl. The increased alveolar size heterogeneity at Pao = 5 cmH_2_O argues for the use of PEEP = 8 cmH_2_O for lung protective mechanical ventilation in this animal model.

## Introduction

Although protective ventilation strategies have been introduced more than 20 years ago, the mortality of acute respiratory distress syndrome (ARDS) patients remains at an unacceptably high level of 30–40%^1^. Mechanical ventilation is a lifesaving therapy but can also aggravate existing lung injury, a phenomenon referred to as ventilation induced lung injury (VILI)^2^. The pathogenic mechanisms include volutrauma, atelectrauma, stress concentration via alveolar interdependence and biotrauma^3^. If lungs are pre-injured and suffer from high surface tension at the onset of mechanical ventilation, the resulting ventilation forces may further aggravate lung injury^4^. Surfactant dysfunction leads to alveolar instability, particularly at low lung volumes. The collapsed alveoli form (micro)atelectasis and can be reopened with increasing pressure during inspiration, a process that potentially stresses the epithelial lining to cause atelectrauma^5^. In addition, as stress concentrators, groups of collapsed alveoli (microatelectases) exert tethering forces on the interalveolar septa of surrounding alveoli that are therefore at risk of overdistension^6^. As a result, heterogenous ventilation is a typical feature of the injured lung, which heightens the risk of developing VILI. If atelectasis is present, the non-atelectatic lung parenchyma (the ‘baby lung’) must absorb the same tidal volume resulting in increased tissue strain which potentiates volutrauma^7^. Hence, alveoli should be kept open throughout the respiratory cycle to avoid harmful reopening processes, stress concentration, and baby lung.

The aim of lung protective ventilation is to adjust the ventilator settings to minimize the risk of VILI. During mechanical ventilation, there are many modifiable parameters such as tidal volume, positive end-expiratory pressure (PEEP), volume and flow rates, and breath frequency^8^. While reduced tidal volume (e.g. 6 ml/kg bodyweight) is established in ARDS patients as a protective ventilation strategy, clinical trials of PEEP optimization are inconclusive^9^. Reduction of tidal volume reduces dynamic strain and thus volutrauma. An ideal PEEP is high enough to minimize cyclic de-recruitment (and thus atelectrauma), the burden of stress concentrators, and the baby lung while it should not be too high to cause impairment of lung perfusion and hyperinflation which can trigger inflammatory response^10^. The load of microatelectases might at least be reduced partially by maintaining an appropriate PEEP. However, both efficiency and pressure ranges needed for recruitability depend on the underlying etiology and pathophysiology^11^. At the alveolar level, three different populations of alveoli have been described during lung injury: (1) One population is characterized by alveolar flooding or irreversible collapse and is not recruitable; (2) a second cohort is unstable and derecruitment can be avoided by appropriate PEEP; (3) the third population appears to be healthy and remains open even at low airway opening pressures^12^. In clinical ARDS, flooded and difficult to recruit alveoli are predominantly found in the dependent lung regions while stable alveoli are in the anterior part of the lung where they are prone to overdistension and thus volutrauma. In between are the unstable alveoli that are subject to atelectrauma and act as stress concentrators and the function of this population can be corrected by appropriate PEEP^13^. Clinical studies using electrical impedance tomography or computed tomography at different airway opening pressures to estimate parenchymal recruitment and overdistension to identify an optimally balanced PEEL-level demonstrated a high heterogeneity in the potential to open distal airspaces between patients^14–17^.

In the present study we investigated the lung-protective potential of alveolar recruitment in the context of parenchymal distension as a function of end expiratory airway opening pressures in an animal model of lung injury. Injury was induced by adenoviral vector mediated overexpression of active TGF-β1 in epithelial cells of the respiratory tract^18,19^. TGF-β1 is important for the development of lung fibrosis but also plays a central role in acute lung injury/VILI where it is up-regulated following high PEEP ventilation and linked with the development of alveolar capillary leakage^20^. During the first seven days after gene transfer to the respiratory system, surfactant proteins are downregulated and surface tension is considerably increased while lung fibrosis is still absent^21–23^. Hence, TGF-β1 overexpression in the lung recapitulates relevant features of lung injury within the first week such as alveolar collapse, vascular leak, and edema formation^19^. Lung mechanical function was measured at different PEEP levels before fixation by vascular perfusion at defined airway opening pressures ranging from 2 to 20 cmH_2_O on expiration after recruitment maneuver. Quantitative morphology based on unbiased stereology was used to measure recruitability and dimensions of acinar airspaces. Lung mechanical properties measured at defined PEEP levels were directly correlated to structural data at corresponding airway opening pressure.

## Methods

### Animal model

Ten-week-old female C57BL/6 mice (Janvier, Le Genest-Saint-Isle, France), were used. The study protocol was carried out in accordance with the Ordinance for Animal Experiments and approved by the LAVES (Lower Saxony State Office for Consumer Protection and Food Safety, approval number: 21/3829) according to the European Animal Welfare Regulations. Hence, all methods were performed in accordance with relevant guidelines and regulations. In addition, the experiments were planned, performed, analyzed, and reported in accordance with the ARRIVE guidelines^24^. After an intraperitoneal (i.p.) anesthesia consisting of Xylazin/Ketamin, (Rompun 2%, Bayer, Leverkusen, Germany and Ketamin, CP-Pharma, Burgdorf, Germany), 10^8^ plaque-forming units of TGF-β1 gene containing adenoviral vectors (AdTGF-β1) diluted in 50 µl PBS were instilled intratracheally. The control group received an empty adenoviral vector (AdCl). Assignment of mice to either group was based on randomization. Seven days after transfection, lung mechanics was measured with the FlexiVent (SCIREQ, Montreal, Canada) before lungs were subjected to either vascular perfusion fixation or broncho-alveolar lavage (BAL).

### Lung mechanics assessment

**Figure 1:**
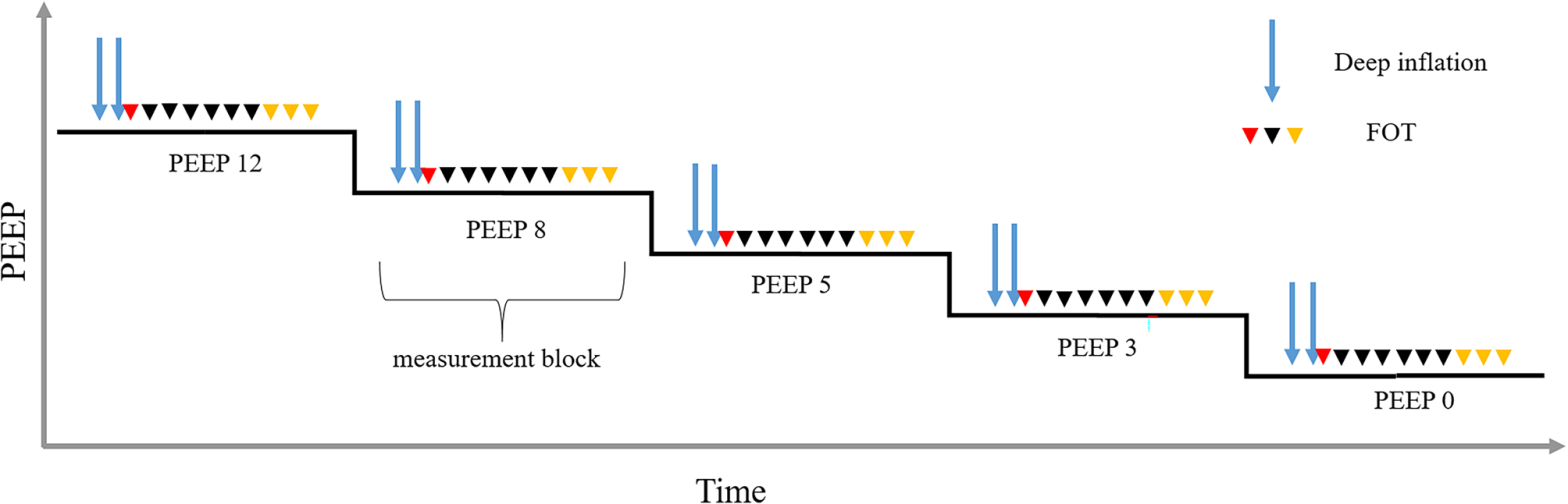
Lung mechanical function assessment. Derecruitability tests were performed during descending PEEP levels from 12 to 0 cmH_2_0. Each derecruitability test (= measurement block) consisted of two deep inflations (blue arrows): the pressure increased from the corresponding PEEP level to a plateau of 30 cmH_2_O and was kept stable for 3 s. Immediately after the second deep inflation the first forced oscillation perturbation (FOT) was performed (red arrowhead). Nine additional FOTs were carried out at intervals of 30 s. Raw data were fit to the constant phase model to calculate tissue damping, tissue elastance and Newtonian resistance. The mean tissue elastance of the last three FOTs (yellow arrow heads) was determined and referred to as H_end_. The difference between H_end_ and the first tissue elastance (red arrowhead) defines ΔH.

Anesthesia of animals was performed with i.p. injection of 80–100 mg/kg Ketamin and 5–7 mg/kg Xylazin diluted in 0.9% NaCl (B.Braun, Melsungen, Germany). In absence of pain reflexes, a tracheotomy was carried out and a cannula (18 G, BD Vacutainer Systems, Plymouth, United Kingdom) was placed into the trachea. The cannula was connected to the FlexiVent, controlled mechanical ventilation started with a respiratory rate of 150 breaths per minute, tidal volume 10 ml/kg and inspiratory-to-expiratory time ratio 1:2. A series of different ventilation maneuvers for lung mechanical measurements were performed based on published methodology^11^. Measurement blocks (derecruitability tests) were carried out as PEEP was sequentially decreased from 12, 8, 5, 3 and finally to 0 cmH_2_O (Fig. 1). Each block started adjusting to the specified PEEP followed by two recruitment maneuvers, in which the airway opening pressure (Pao) increased continuously over a ramp of 3 s to 30 cmH_2_O and was then kept stable for 3 s (= deep inflation). The inspiratory capacity, which is the volume of air displaced into the lung from the corresponding PEEP level to Pao = 30 cmH_2_O was recorded. While continuing ventilation with the defined PEEP level, forced oscillation technique (FOT, Quick-Prime3 of the flexiVent ventilator software) measurements with an onset pressure identical to the PEEP level were repetitively applied to measure impedance spectra. The first FOT was carried out 2 s after the recruitment maneuver to reflect the situation of optimal recruitment. Further FOT followed in intervals of 30 s. over a period of 4.5 min. The constant phase model^25^ was fit to impedance spectra to obtain tissue elastance (H), tissue damping (G), Newtonian resistance (Rn) and the hysteresivity (G/H)^26^. The difference between the mean of the last three tissue elastance values (= H_end_) and the first tissue elastance value of a derecruitability test is referred to as ΔH. If the coefficient of determination (COD), which describes the goodness of fitting to the raw data, was below 0.8 the measurement was excluded from further analysis. At the end of the measurement sequence pressure–volume curves were recorded by increasing pressure stepwise from 0 to 30 cmH_2_O followed by a corresponding stepwise decrease to 0 cmH_2_O. Quasi static pressure and volume data were used to calculate quasi-static compliance as the slope of the pressure–volume curve at Pao = 5 cmH_2_O on expiration. In a subset of animals, the dynamic compliance during PEEP = 8 cmH_2_O ventilation was determined based on the single compartment model using the SnapShot perturbation of the FlexiVent system.

### Fixation, sampling and tissue processing

At the conclusion of ventilation a midline laparotomy and thoracotomy was performed during PEEP = 2 cmH_2_O ventilation and a tidal volume of 10 ml. After opening the chest, the rib cage was strut apart so that the lung and the heart were freely visible and two deep inflations were performed. A custom fixation perturbation was then applied consisting of a 6-s ramp to 30 cmH_2_O, a 3-s hold and a slow decrease to either 20, 10, 5 or 2 cmH_2_O. In mice, lung volumes for airway opening pressures of 10, 5 and 2 cmH_2_O of an open chest were found to be comparable to lung volumes at airway opening pressures of 12, 5 and 0 cmH_2_O of a closed chest, respectively^27^. Hence, stereological data from lungs fixed at Pao 10, 5 and 2 cmH_2_O could be correlated to lung mechanical data measured at intact chest wall at PEEP = 12, 5 and 0 cmH_2_0, respectively. At a stable pressure and zero airflow, the trachea was ligated and a vascular perfusion fixation carried out. A cannula (23G, BD Vacutainer Systems, Plymouth, United Kingdom) was inserted into the right ventricle, the left atrium incised and the pulmonary vasculature rinsed with 0.9% NaCl/heparin (25,000 IU/ml, Ratiopharm, Ulm, Germany) with a ratio of 400:1 at a hydrostatic pressure of 30 cmH_2_O. After the lung whitened, fixation was performed with a solution of 1.5% glutaraldehyde, 1.5% paraformaldehyde in 0.15 M HEPES buffer. Finally, the lungs were stored in this solution for more than 24 h at 4 °C. Lungs that were damaged during dissection process and those where the alveolar capillary network was not open, indicating poor fixation quality, were excluded from further morphological analysis.

**Figure 2:**
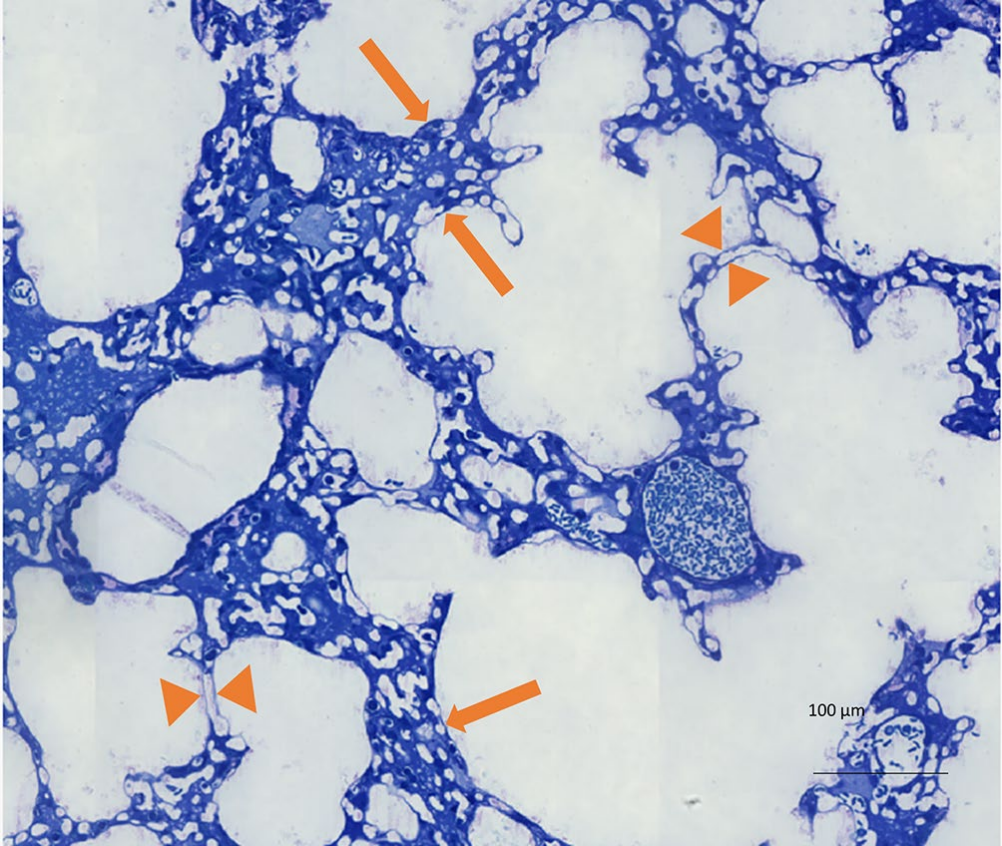
Examples of collapsed septa (arrows) and open septa (arrowheads). This lung was fixed at a pressure of 5 cmH_2_O.

After dissection of the lung, the volume was determined by means of water displacement according to Archimede’s principle^28^. Sampling, embedding, sectioning and staining was performed as published previously^28,29^. In brief, systematic uniform random sampling based on the smooth fractionator was performed so that the obtained tissue samples were representative for the whole organ^30^. The lungs were embedded in agar and cut in 1 mm thick slices. These slices were sorted by size and every second slice was used for light microscopy. The samples were embedded in Technovit 8100 (Kulzer Heraeus, Wehrheim, Germany) and cut in 1.5 µm sections. The 1st and the 4th section of a consecutive series of sections were mounted on a glass slide and stained with Toluidine blue for light microscopy. The distance from the top of the first to the top of the fourth section was 4.5 µm and corresponded to the disector height used for determination of the number of open alveoli (see below). The light microscopic sections were scanned with an AxioScan (Zeiss, Oberkochen, Germany) using a primary magnification of 20×. After that, the whole slides were analyzed by design-based stereology. At least four sections per lung were loaded into the newCast software (Visiopharm, Hoersholm, Denmark) for systematic uniform random area sampling to yield 100–200 randomized images per lung for stereological analysis. The overall goal was to obtain at least 100 counting events per stereological parameter^31^. Test points, overlaid on the sampled images were used to determine volume fractions of structures of interests within the reference space. The total lung volume was divided into two categories, the non-parenchyma, and the parenchyma. The parenchyma was defined as all structures usually involved in gas exchange and included alveolar and ductal airspaces as well as the interalveolar septa. Occasionally, traces of intra-alveolar edema were suspected to intersperse between collapsed septa and could not be safely delineated at light microscopic level. Thus, it was not counted separately. The interalveolar septa were further differentiated into two fractions: recruited (= unfolded septa) and derecruited (= folded septa). As folded septa implies that the corresponding alveolar airspaces are partly or completely collapsed, we also refer to folded septa as collapsed septa. An example of these different types of septa is shown in Fig. 2. Recruited septa were defined by a single layer of alveolar capillary network. Collapsed septa could be identified by the existence of more than one layer of the alveolar capillary network^32^. Points hitting the structure of interest (e.g. alveolar airspace) were than divided by the sum of points hitting the reference space (e.g. lung parenchyma) so that volume fractions were calculated and converted to absolute data per organ by multiplication of volume fractions with the reference space.

Test lines were used to determine the surface area of acinar airspaces by intersection counting. In addition, the arithmetic mean septal wall thickness was calculated as volume-to-surface ratio of interalveolar septa. The number of open alveoli per lung was estimated using the physical disector method on two corresponding sections separated by a distance of 4.5 µm (= disector height). A counting frame was projected onto corresponding fields of view from the two sections. Alveoli, which were open to the alveolar duct on one image but not on the other, were counted. The area of the counting frames multiplied with the disector height resulted in the disector volume in which open alveoli were counted, so that the numerical density of alveoli within lung parenchyma could be calculated and converted into the absolute number of open alveoli per lung. The number-weighted mean volume of alveoli was calculated as the quotient of the absolute volume of alveolar airspaces and the total number of alveoli per lung. The volume-weighted mean alveolar volume was determined by using the point sampled intercept method (PSI)^23^. Test points were projected on randomized field of view to sample alveoli for calculation of their volumes. Since larger alveoli had a higher probability to be hit (and sampled) by the test points, the determination of volume-weighted mean volume of alveoli favored larger alveoli. Hence, the volume-weighted mean volume did not only depend on the number-weighted mean alveolar volume but also the variability of alveolar sizes, e.g. the coefficient of variation (CV^2^(V_X_(alv))^33,34^. The later was calculated from number and volume weighted mean alveolar volume according to the formula given in Table 1. In addition, the heterogeneity of the dimensions of acinar airspaces was investigated by direct measurements of chord lengths^35^. For measuring the chord length, a random test line overlaying the sections was used. If the line intersected a septum, the length to the next intersection with the septum was measured. Its distribution provided information about the one-dimensional strain and heterogeneity of acinar airspaces. Table 1 summarizes the stereological parameters.

**Table 1:** Definition of stereological parameters.

Parameter	Abbreviation	Determination	Magnification
Lung volume	V(lung)	Fluid displacement	
Absolute volume of non-parenchyma per lung	V(nopar,lung)	Point counting	10×
Absolute volume of parenchyma per lung	V(par,lung)	Point counting	10×
Absolute volume of alveolar airspace in lung parenchyma	V(alv,par)	Point counting	10×
Absolute volume of ductal airspace in lung parenchyma	V(duc,par)	Point counting	10×
Absolute volume of septal walls in lung parenchyma	V(sep,par)	Point counting	10×
Absolute volume of recruited septal walls in lung parenchyma	V(recsep,par)	Point counting	10×
Absolute alveolar surface area in lung parenchyma	S(alv,par)	Intersection counting	10×
Alveolar surface density within lung parenchyma	S_V_(alv,par)	Intersection counting	10×
Fraction of collapsed septa	collapsed sep	Point counting	10×
Arithmetic mean thickness of septal wall (volume-to-surface ratio)	τ(sep)	= 2*V(recsep,par)/S(alv,par)	
Absolute number of open alveoli in lung parenchyma	N(alv,par)	Physical disector	20×
Numerical density of alveoli within lung parenchyma	N_V_(alv,par)	Physical disector	20×
Number-weighted mean volume of alveoli	V_N_(alv)	= V(alv,par)/N(alv,par)	
Volume-weighted mean volume of alveoli	V_V_(alv)	Point sampled intercepts	20×
Chord length	Chord length	Distance measurements	10×
Coefficient of variation of alveolar size distribution	CV^2^(V_x_(alv))	= V_v_(alv)/V_N_(alv) − 1	

### Biochemical analysis

After the FlexiVent measurements, a bronchoalveolar lavage (BAL) was performed on four animals from the AdCl and the AdTGF-β1 group. The pulmonary vasculature was rinsed with 0.9% NaCl water solution and then 1 ml 0.9% NaCl water was instilled and withdrawn via the trachea three times. These lavages were examined for both total protein content and albumin content. The protein content was measured with the Pierce™ BCA Protein Assay Kit (Thermo Fisher, Waltham, USA) and the albumin content with the Mouse Albumin ELISA Kit E99-134 (Bethyl, Montgomery, USA) according to manufacturers’ instructions. In addition, the lavage was centrifuged for 10 min with 1500 U/min at 4 °C, stained with trypan blue dye and then 50,000 cells of this suspension were spun onto cytospots for further differentiation.

### Statistical analysis

The statistical analysis of the lung mechanical and the stereological data was carried out with GraphPad Prism (Version 10, Graphpad Software, Inc., Bosten, USA). For statistical significance, a two-way ANOVA on ranks taking the factors “treatment” (AdCl vs TGF-β1) and “pressure” (PEEP and Pao, respectively) and the interaction of these factors into consideration. If significant factor effects were seen, a multiple comparison test was performed with an adjustment of the p-level with the Sidak correction. For correlation analyses Pearson (for normally distributed data) or Spearman tests were performed. The group means of the chord lengths measurements was compared between AdCl and AdTGF-β1 at a given Pao with a non-parametric rank sum test (Mann–Whitney U-test). Frequency distributions of chord lengths were compared for equality using Kolmogorov–Smirnov Test. Density distribution functions of chord lengths were generated using Kernel functions (Matlab R2023a, MathWorks, Natick, Massachusetts, USA). The p-level of statistically significant differences was < 0.05.

## Results

### Lung mechanical function

**Figure 3:**
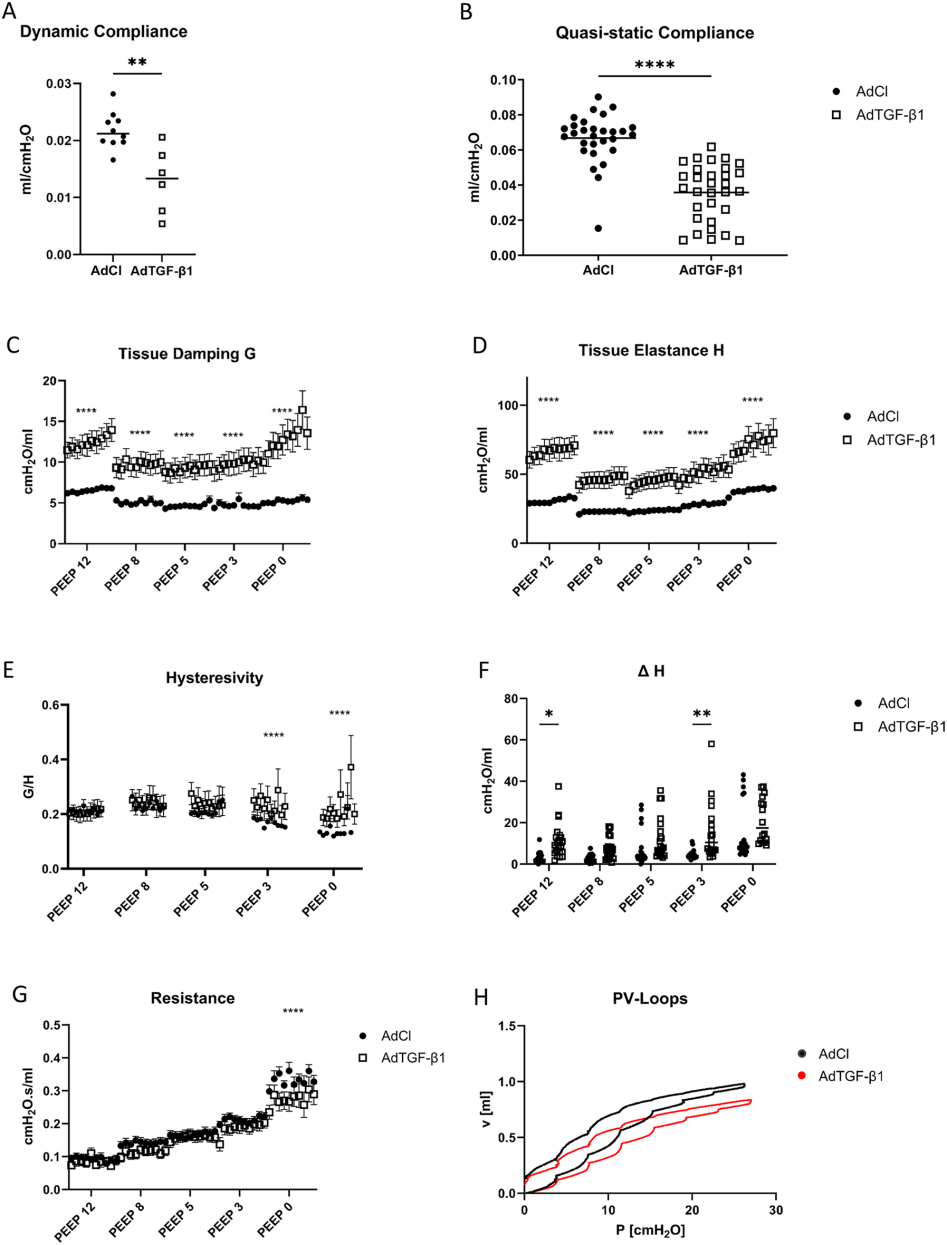
Lung mechanical function. The dynamic compliance was measured by single-frequency (SnapShot) maneuvers during PEEP = 8 cmH_2_O ventilation in a small cohort and was significantly reduced in AdTGF-β1 (**A**). The quasi-static compliance showed significant differences between the AdCl and the AdTGF-β1 groups (**B**). (**C**–**G**) show parameters calculated from forced oscillation technique (FOT) measurements fit to constant phase model. Data were determined by derecruitability tests during mechanical ventilation with descending PEEP-levels. Tissue damping (**C**), tissue elastance (**D**), hysteresivity (the quotient of G/H) (**E**), increase in tissue elastance after recruitment (ΔH = difference between the mean of the last three and the first tissue elastance value) (**F**), and Newtonian Resistance (**G**) are shown. Quasi-static pressure–volume loops are illustrated in H (mean of n = 10 per group). In (**C**, **D**, **E**, **G**) the mean and standard error of the mean is given. Differences between AdTGF-β1 and AdCl are indicated as follows: *p ≤ 0.05, **p ≤ 0.01, ****p ≤ 0.0001.

The tissue elastance (H), tissue damping (G) and compliance (quasi-static and dynamic) were significantly worse in the AdTGF-β1 group independent of the PEEP level (Fig. 3A–D). Over the range of investigated PEEPs, both the tissue elastance H and the tissue damping G showed a significant increase in the AdTGF-β1 animals compared to AdCl. This increase demonstrated the stiffness of the diseased lungs (H) and the inability of the tissue to release the energy received (G). The fact that the AdTGF-β1 lungs have lost their elastic properties is further supported by the decreased quasi-static and the dynamic compliances (Fig. 3A,B). The PEEP levels also had significant effects on lung mechanical properties (Fig. 3C,D). Minimal tissue elastance as well as tissue damping was noticed in the mid parts of investigated PEEP levels, e.g., 5 and 8 cmH_2_O. Conversely, either large PEEP = 12 cmH_2_O or low PEEP = 0 cmH_2_O demonstrated much higher H and G. The increase in H with larger PEEP can be explained by progressive straightening of collagen fibrils resulting in strain-stiffening of lung parenchyma. Except for low PEEP ventilation (PEEP = 0 and 3 cmH_2_O), the hysteresivity did not show significant differences between the AdCl and the TGF-β1 animals (Fig. 3E). This means that G and H show comparable relative changes in both AdTGF-β1 and AdCl during ventilation with a PEEP > 3 cmH_2_O, so that quotient of G and H nearly stays the same. ΔH, a marker of progressive derecruitment of distal airspaces during the derecruitability test, is calculated as the difference between the mean of the last three (H_end_) and the first measurements of H after recruitment, and shows inter-group significances in the PEEP 12 and the PEEP 3 measurements (Fig. 3F). The resistance showed a significant increase in AdTGF-β1 compared to AdCl group at PEEP = 0 cmH_2_O. Moreover, a significant PEEP effect could be observed in both groups: with decreasing PEEP-level Rn increased (Fig. 3G). The inspiratory capacity and slope of the quasi-static pressure–volume loop was reduced in AdTGF-β1 compared to AdCl (Fig. 3H).

### Morphology and morphometry

**Figure 4:**
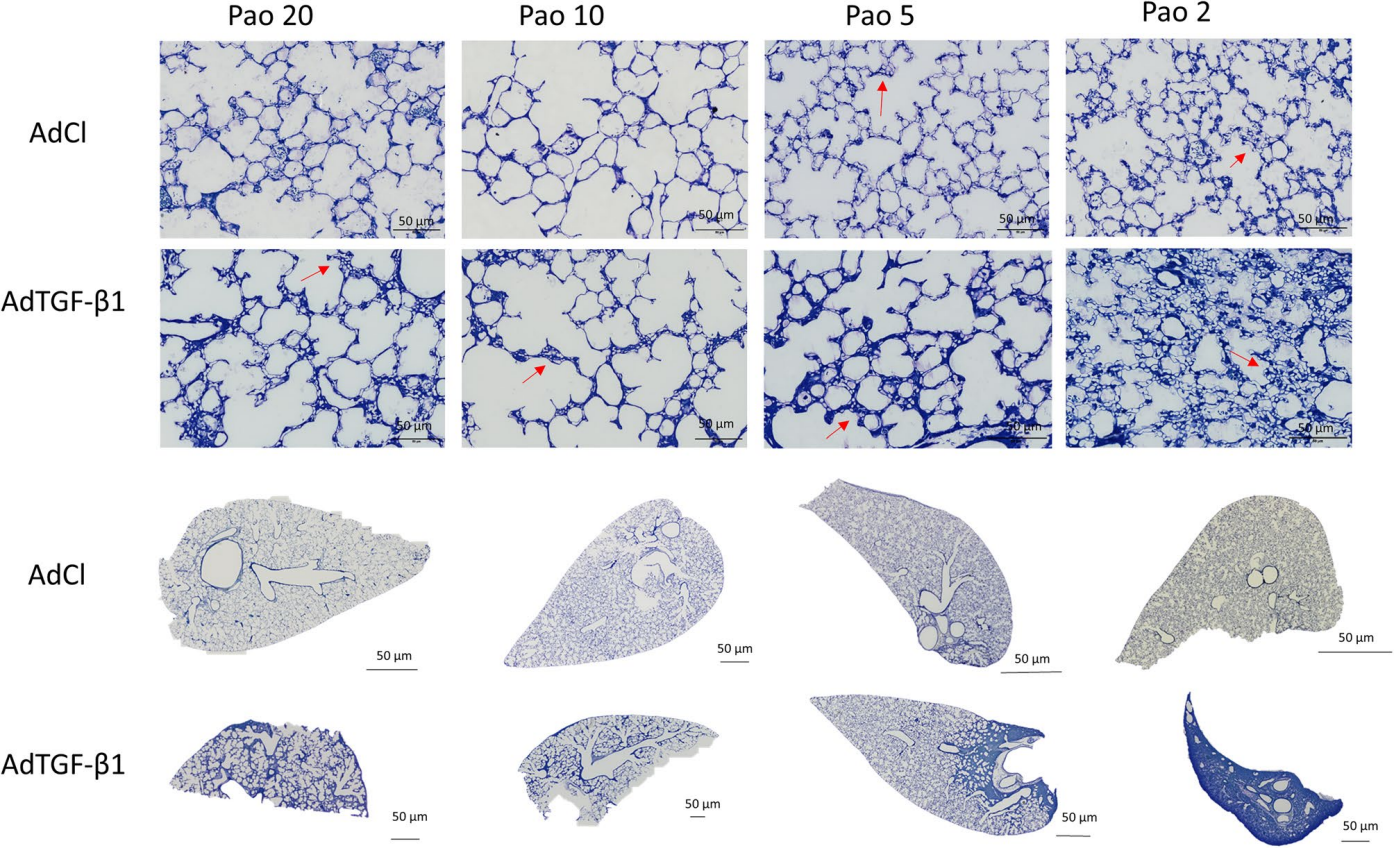
Morphological differences. The light microscopical sections stained with toluidine blue highlight the structural changes between the both groups (AdCl and AdTGF-β1) and also between the different airway opening pressures (Pao) during vascular perfusion. With the decline of the Pao, the more atelectasis (red arrows) can be seen.

The morphology at light microscopic level clearly differed between both study groups and the various Pao at fixation (Fig. 4). The AdTGF-β1 group showed more microatelectases and collapsed septa due to alveolar instability. These changes were already visible on the low-magnification overview images of the slides (bottom row of Fig. 4) which also revealed a remarkable heterogeneity of pathological alterations within the lung parenchyma. The collapsed septa (blue regions) were irregularly distributed over the sections. Higher magnification revealed septal walls including alveolar capillary networks, in most instances open and free of blood cells as a result of vascular perfusion fixation. With an optimal perfusion fixation of the lung, the microatelectases were easily identified. In both the AdTGF-β1 and the AdCl groups there were septa piled up and folded on each other which we referred to as collapsed septa. However, in the AdTGF-β1 group the density of these collapsed regions was much higher and appeared to decline with an increase in Pao. The thickened septal walls were observed in concert with a rarefication of alveolar airspaces. Overall, there was very little edema and inflammatory infiltrate observed in the AdTGF-β1. Small regions of alveolar edema were seen, predominantly close to larger vessels and conducting airways.

**Figure 5:**
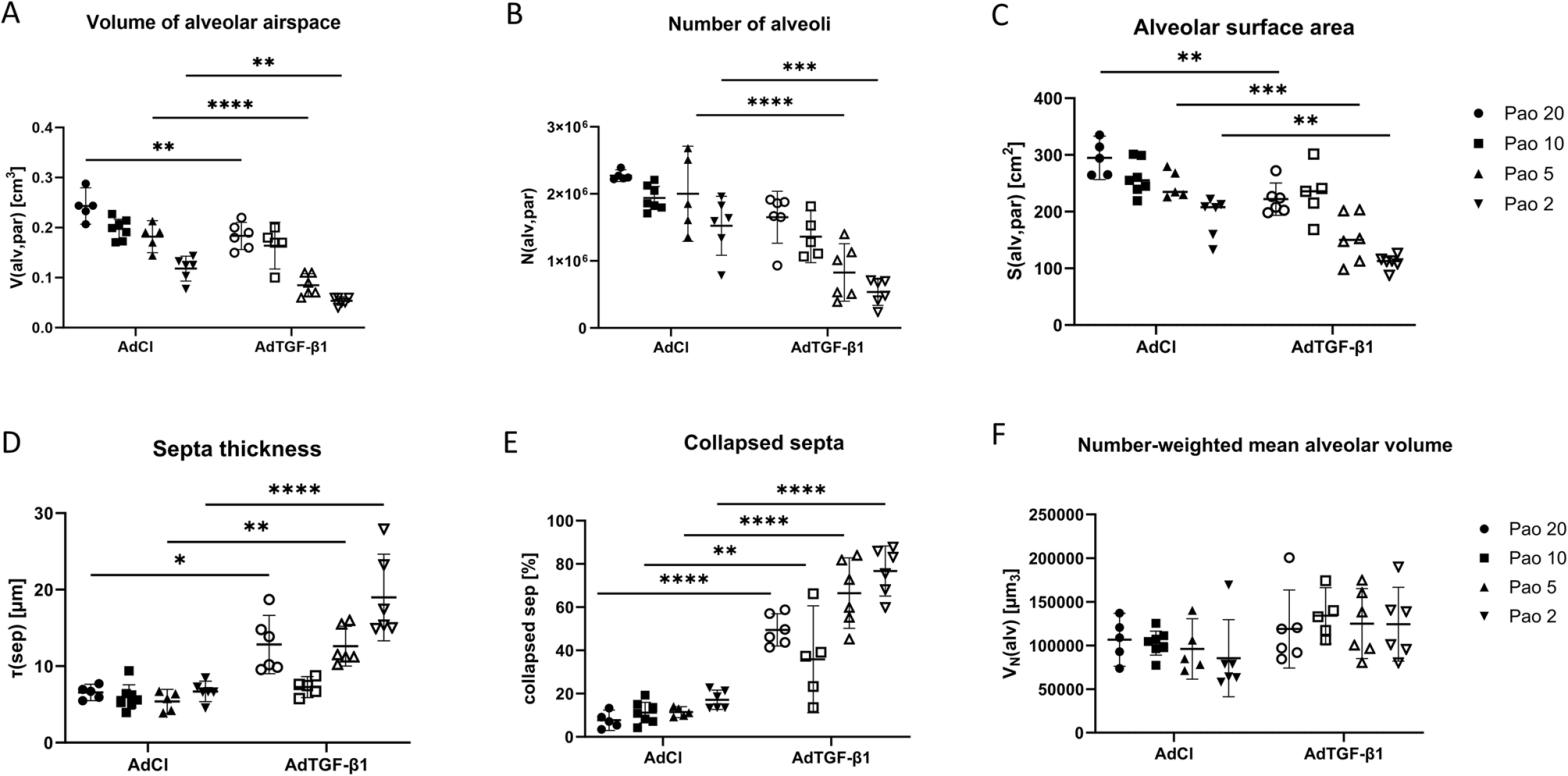
Lung stereology. The alveolar airspace within the parenchyma (V(alv,par)) shows significant differences between the AdCl and the AdTGF-β1 animals (**A**). The number of open alveoli (N(alv,par)) (**B**) and the alveolar surface within the parenchyma (S(alv,par)) (**C**) show significances in all fixation groups except the Pao = 10 cmH_2_O. The septa thickness and the fraction of collapsed septa are increased in AdTGF-β1 compared to AdCl (**D**, **E**). In AdCl septa thickness remained roughly stable while it decreased in AdTGF-β1 with increasing Pao (**D**). Fraction of collapsed septa decreased in both groups with higher Pao (**E**). The average alveolar volume (V_N_(alv)) shows clearly smaller difference between both groups (**F**). (*p ≤ 0.05, **p ≤ 0.01, ***p ≤ 0.001, ****p ≤ 0.0001).

Stereological data are summarized in Table 2 and visualized in Fig. 5. The total lung and non-parenchyma volumes were not statistically significant different between AdTGF-β1 and AdCl. While the total lung volume showed a clear dependence on the Pao, the non-parenchyma volume did not. The volume of lung parenchyma, on the other hand, depended on both the Pao and the treatment. While at lower Pao ranging from 2 to 10 cmH_2_O no significant differences were observed between AdTGF-β1 and AdCl, the volume of lung parenchyma was significantly reduced in AdTGF-β1 compared to AdCl at Pao = 20 cmH_2_O. The composition of the lung parenchyma differed considerably between study groups at the varying Pao. Compared to AdCl, the volume of the alveolar airspaces within the parenchyma was decreased in the AdTGF-β1 lungs at Pao = 2 and 5 cmH_2_O (Fig. 5A). The volume of the ductal airspaces remained unchanged in AdTGF-β1 compared to AdCl over the range of considered Pao so that the ratio between the alveolar and the ductal airspace differed between the groups at Pao = 2 and 5 cmH_2_O. These findings correlated with the number of open alveoli, which was significantly decreased in the AdTGF-β1 animals. In the AdCl group, there are 1.3 times more alveoli at a pressure of 20 cmH_2_O compared to the pressure at 2 cmH_2_O. In the AdTGF-β1 animals, the number of alveoli is three times higher at a pressure of 20 cmH_2_O than at 2 cmH_2_O (Fig. 5B). According to the 2-way ANOVA, TGF-β1 gene-transfer had a significant effect on the mean alveolar size (= number-weighted mean alveolar volume), which was most pronounced at Pao = 2 and 5 cmH_2_O where AdTGF-β1 trended larger compared to AdCl (Fig. 5F). Moreover, both the treatment and the Pao had significant effects on the volume-weighted mean alveolar volume (Table 2). With increasing pressure, the volume-weighted mean alveolar pressure increased and at lower Pao the volume-weighted mean alveolar volume was significantly larger in AdTGF-β1 compared to AdCl. This finding resulted partly from an increased alveolar size heterogeneity in AdTGF-β1 as indicated by a larger coefficient of variation (CV^2^(V_X_(alv)) (Table 2). Hence, in AdTGF-β1 there was increased alveolar size heterogeneity at Pao = 2—5 cmH_2_O in addition to the increased number-weighted mean alveolar volume. The alveolar surface area shows significant differences between both groups as well (Fig. 5C). The amount of collapsed septa as well as the septa thickness is increased in the AdTGF-β1 group. (Fig. 5D,E). All these parameters show that the alveolar instability is increased during TGF-β1 overexpression.

**Table 2:** Stereological data (mean and standard deviation) and p-values for the factors “Pao” and “treatment” as well as the interaction between both of them are shown. The asterisks mark adjusted p-levels for a given Pao vs. AdCl group as follows: *p ≤ 0.05, **p ≤ 0.01, ***p ≤ 0.001, ****p ≤ 0.0001.

	AdCl				AdTGF-β1				p-values		
	Pao 20	Pao 10	Pao 5	Pao 2	Pao 20	Pao 10	Pao 5	Pao 2	Treatment	Pressure	Interaction
V(lung) cm^3^	0.79 (0.12)	0.67 (0.05)	0.50 (0.03)	0.38 (0.06)	0.70 (0.11)	0.66 (0.06)	0.43 (0.06)	0.39 (0.05)	0.1496	< 0.0001	0.7361
V(nopar,lung) cm^3^	0.10 (0.03)	0.11 (0.04)	0.08 (0.02)	0.08 (0.01)	0.09 (0.04)	0.14 (0.03)	0.10 (0.02)	0.13 (0.01)	0.0514	0.0710	0.2828
V(par,lung) cm^3^	0.69 (0.11)	0.55 (0.04)	0.42 (0.04)	0.30 (0.06)	0.61 (0.07)*	0.52 (0.05)	0.32 (0.05)	0.26 (0.05)	0.0013	< 0.0001	0.3084
V(alv,par) cm^3^	0.24 (0.05)	0.20 (0.02)	0.18 (0.02)	0.12 (0.12)	0.18 (0.02)**	0.17 (0.03)	0.08 (0.02)***	0.05 (0.01)*	< 0.0001	< 0.0001	0.0381
V(duc,par) cm^3^	0.35 (0.06)	0.28 (0.03)	0.17 (0.02)	0.12 (0.03)	0.30 (0.06)*	0.27 (0.03)	0.14 (0.03)	0.11 (0.02)	0.0096	< 0.0001	0.0709
V(alv,par)/ V(duc,par)	0.69 (0.07)	0.71 (0.10)	1.09 (0.12)	0.96 (0.08)	0.62 (0.08)	0.62 (0.16)	0.62 (0.12)****	0.48 (0.04)****	< 0.0001	0.0006	< 0.0001
S(alv,par) cm^2^	345.5 (68.5)	259.9 (28.2)	247.9 (21.2)	196.5 (32.2)	217.4 (12.8)**	232.3 (42.97)	152.6 (39.9)***	110.6 (11.7)**	< 0.0001	< 0.0001	0.1008
S_V_(alv,par) 1/cm	432.7 (51.6)	469.3 (38.8)	596.2 (32.9)	628.0 (79.8)	355.8 (29.3)	443.7 (53.8)	477.8 (64.0)*	427.0 (49.7)****	< 0.0001	< 0.0001	0.0058
V(recsep,par) cm^3^	0.09 (0.01)	0.06 (0.01)	0.06 (0.01)	0.05 (0.01)	0.07 (0.02)	0.05 (0.02)	0.03 (0.02)	0.02 (0.01)	0.0001	< 0.0001	0.6597
V(sep,par) cm^3^	0.1 (0.01)	0.07 (0.009)	0.07 (0.01)	0.06 (0.01)	0.14 (0.03)*	0.09 (0.01)	0.1 (0.02)	0.12 (0.02)***	< 0.0001	0.0003	0.1181
collapsed sep %	7.68 (3.39)	11.20 (4.78)	11.44 (1.83)	17.90 (3.85)	49.49 (6.48)****	35.90 (17.84)**	66.49 (14.15)****	76.72 (10.06)****	< 0.0001	< 0.0001	0.0009
τ(sep) µm	6.56 (0.78)	5.97 (1.58)	5.39 (1.14)	6.70 (1.27)	12.83 (3.31)	7.26 (0.98)	12.61 (2.26)***	18.98 (4.93)****	< 0.0001	< 0.0001	0.0003
N(alv,par) 10^6^	2.27 (0.06)	1.94 (0.17)	2.00 (0.51)	1.67 (0.38)	1.65 (0.34)	1.36 (0.28)	0.83 (0.37)****	0.54 (0.17)***	< 0.0001	< 0.0001	0.1340
N_V_(alv) 10^6^/cm^3^	3.38 (0.61)	3.51 (0.42)	4.79 (1.04)	5.12 (1.37)	2.66 (0.60)	2.38 (0.35)	2.32 (0.78)***	1.73 (0.56)****	< 0.0001	0.2908	0.0026
V_N_(alv) 10^3^µm^3^	106.5 (21.9)	102.6 (13.7)	90.3 (26.0)	68.5 (38.4)	118.8 (38.9)	134.0 (23.2)	125.0 (34.9)	124.3 (36.7)	0.0080	0.8041	0.8125
V_V_(alv) 10^3^µm^3^	253.6 (68.8)	141.1 (20.6)	100.1 (41.2)	103.0 (46.1)	247.2 (86.1)	172.8 (31.3)	197.7 (43.4)	188.9 (34.9)*	0.0007	< 0.0001	0.0399
CV^2^(v_X_(alv))	1.36 (0.41)	0.41 (0.39)	0.12 (0.12)	0.21 (0.13)	1.12 (0.56)	0.29 (0.16)	0.63 (0.27)	0.61 (0.46)	0.0052	0.0002	0.1552

**Figure 6:**
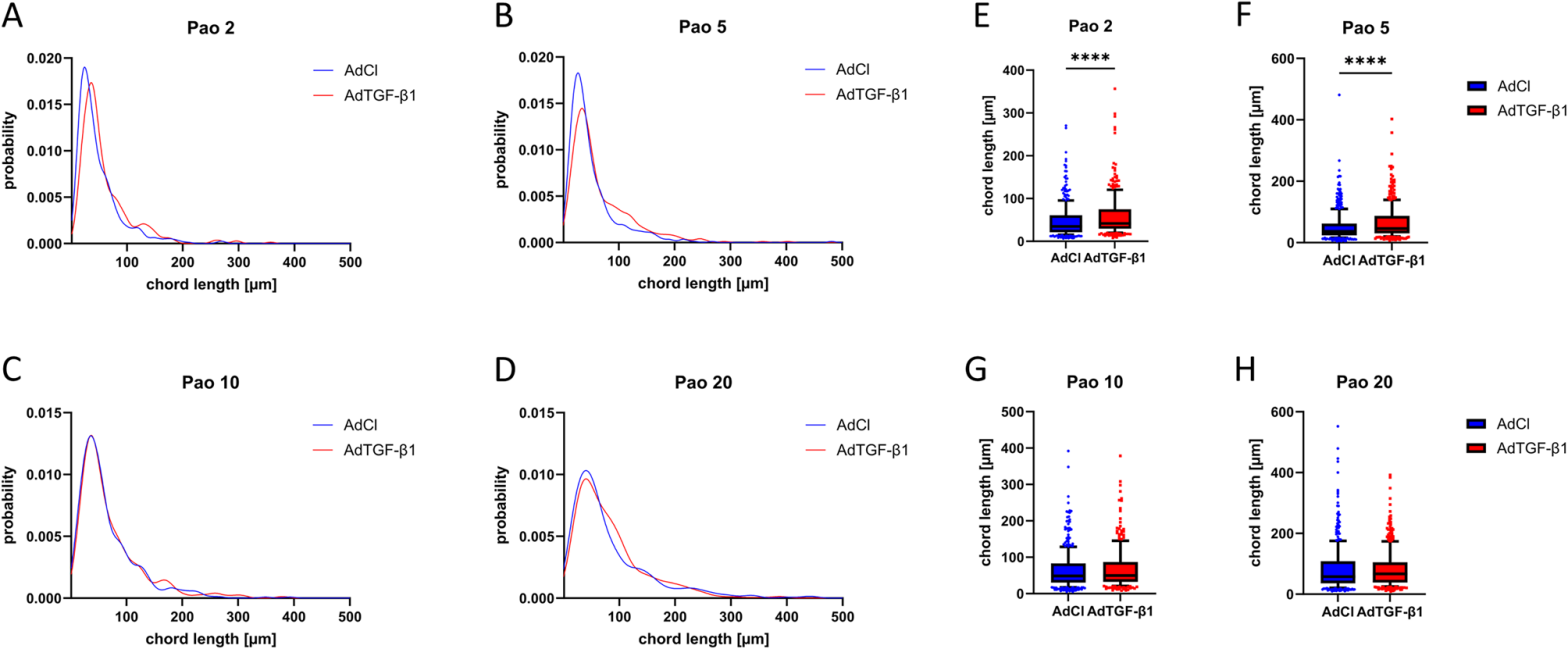
Heterogeneity of distal airspaces. Through the chord lengths measurement, size distributions of distal airspaces and thus heterogeneity were investigated. The graphs (**A**–**D**) show the kernel density distribution function of the acinar chord lengths and graphs (**E**–**H**) the corresponding box plots. The graphs of the animals fixed with a Pao of 10 cmH_2_O are very similar and show nearly the same distribution/heterogeneity in AdCl and AdTGF-β1. The other fixation groups show more variation between the groups. (****p ≤ 0.0001).

To quantify heterogeneity and distension of distal airspaces, comprised of the alveolar and duct airspaces together, chord lengths measurements were performed. Figure 6 shows the density distribution functions and the box plots of chord length measurements for the different Pao levels. The chord lengths distributions differed in Pao 2, 5 and 20 cmH_2_O between AdCl and AdTGF-β1. At Pao = 2 and 5 cmH_2_O the frequency peak of chord length was right shifted and chord lengths ranging between approximately 100 to 150 µm tended to be more often measured in AdTGF-β1 compared to AdCl. The density distribution functions of the chord lengths of the Pao = 10 cmH_2_O fixed lungs was almost identical in AdCl and AdTGF-β1 (Fig. 6C). The lungs fixed at Pao = 20 cmH_2_O showed a broadening of the density distribution function in the AdTGF-β1 animals compared to AdCl (Fig. 6D). Similar results were reflected in the box plots, where there was no significant difference at the Pao 10 cmH_2_O fixed lungs in contrast to the differences observed at lower pressures.

### Collapsed septa and implications to alveolar micromechanics

**Figure 7:**
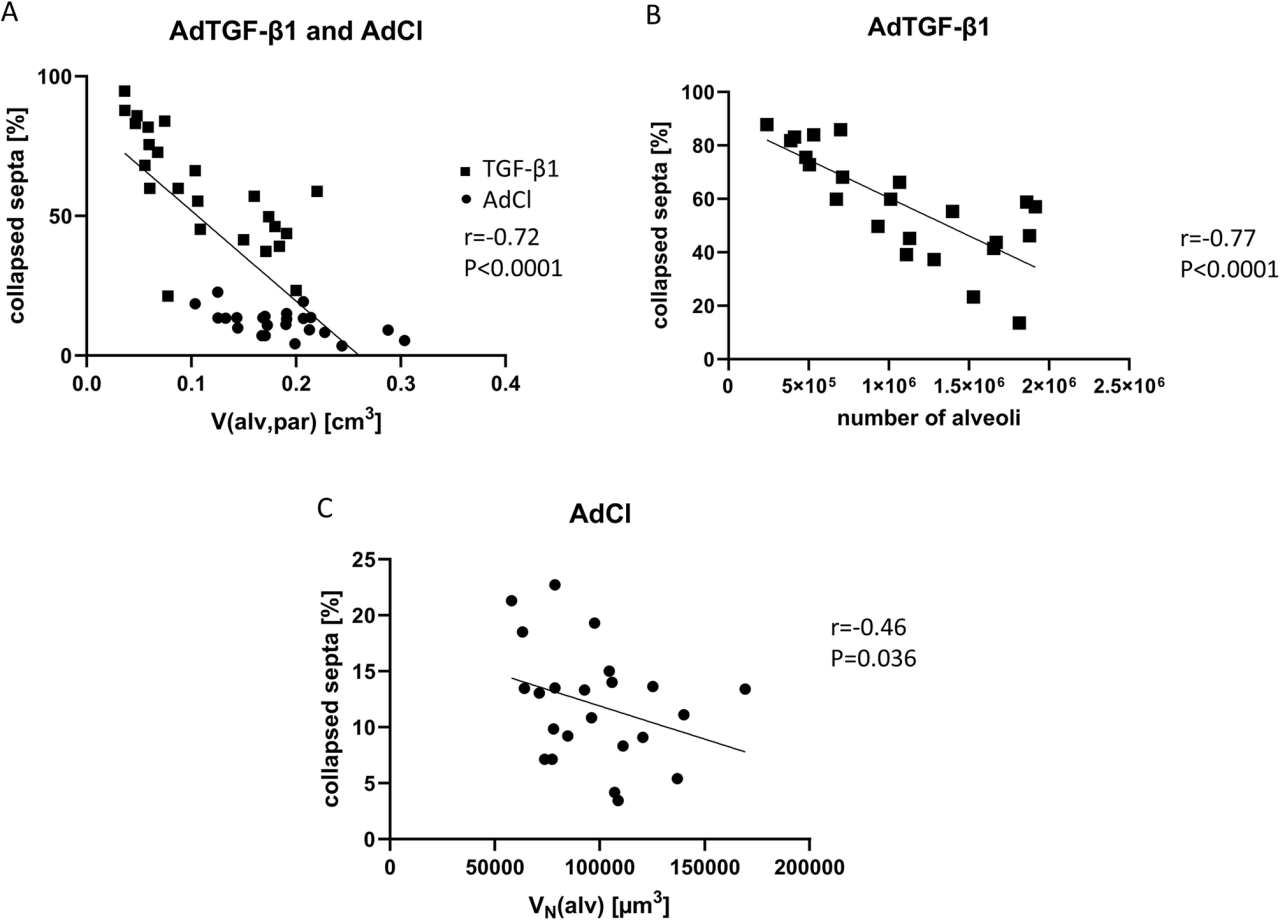
Correlation of the structural data. A strong inverse correlation was established between the volume of alveolar airspaces (V(alv,par)) and collapsed septa in pooled data of groups AdTGF-β1 and AdCl (**A**). In AdTGB-β1 the number of open alveoli strongly correlated with the fraction of collapsed septa (**B**). In AdCl a weak correlation (− 0.46) between number weighted mean alveolar volume (V_N_(alv) and collapsed septa was found (**C**).

Correlation analyses were performed to investigate the association between collapsed septa and alveolar micromechanics. With decreasing Pao, the percentage of collapsed septa increased in both the AdCl and AdTGF-β1 treated lungs. This increase was strongly inversely correlated with the total volume of alveolar airspaces (Fig. 7A, r = − 0.72). In AdTGF-β1 mice the fraction of collapsed septa was a good surrogate for the decrease in the total number of open alveoli as indicated by a strong negative correlation between these two parameters (Fig. 7B, r = − 0.77). In AdCl treated mice, the increasing appearance of collapsed septa correlated negatively with progressive decrease in number-weighted mean alveolar volume. However, this correlation was weak with a low negative slope (r =  − 0.46, p = 0.036).

### Structure-function relationship

**Figure 8:**
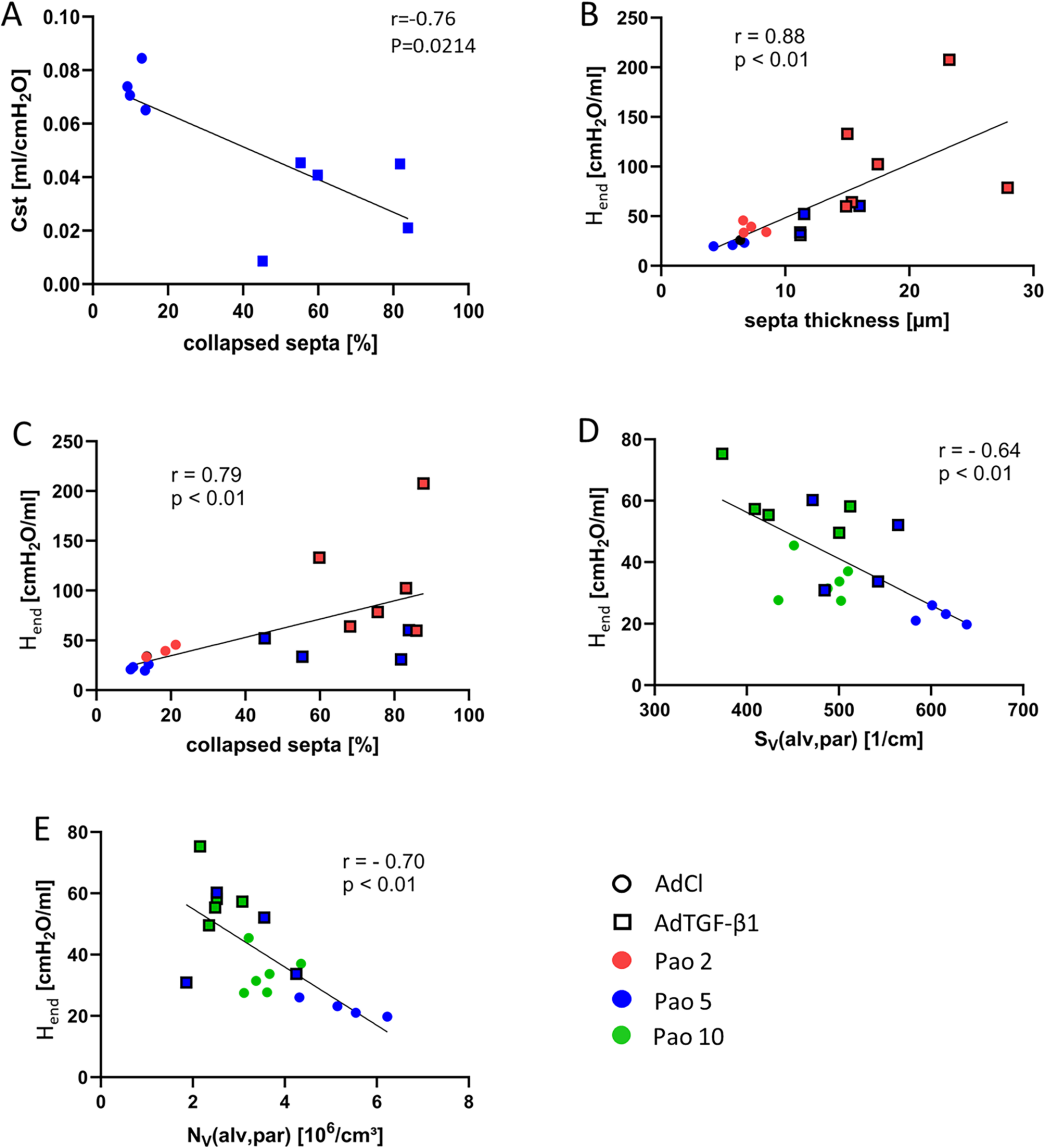
Correlation of lung mechanic and the structural data. The quasi-static compliance and collapsed septa of the Pao 5 groups show a strong correlation (**A**). The increase in tissue elastance with decreasing PEEP from 5 and 0 cmH_2_O strongly correlated with the thickness of interalveolar septa (**B**) and fraction of collapsed septa (**C**) at corresponding airway opening pressure of Pao 5 and 0 cmH_2_O during vascular perfusion fixation. The increase in H_end_ with elevating PEEP ventilation from 5 to 12 cmH_2_O in AdCl and AdTGF-β_1_ correlated with the alveolar surface area density S_V_(alv,par) (**D**) and the alveolar number density N_V_(alv,par) (**E**).

The quasi-static compliance Cst is measured by the flexiWare ventilator software as the slope of the pressure–volume relationship at an airway opening pressure of 5 cmH_2_O on expiration and was significantly lower in AdTGF-β1 compared to AdCl. Accordingly, the structural parameters in AdCl and AdTGF-β1 determined at Pao = 5 cmH_2_O on expiration were correlated to Cst. Among structural parameters the fraction of collapsed septa showed the strongest negative correlation to Cst: the more septa were collapsed, the lower was the Cst (Fig. 8A, r = − 0.76). Measurements of tissue elastance were performed repeatedly after two recruitment maneuvers for up to 4.5 min and. Tissue elastance values were minimized in both AdTGF-β1 and AdCl at PEEP = 5–8 cmH_2_O and increased with lower and larger PEEP levels (Fig. 3D). The increase in H_end_ (the mean of the last three tissue elastance values of a derecruiability test) between PEEP 5 and 0 cmH_2_O was highly correlated to an increase in the mean thickness of septal walls (τ(sep)) (Fig. 8B, r = 0.88) and fraction of collapsed septa (Fig. 8C, r = 0.79). The structural parameters that correlated with these mechanical properties were partly different in AdCl and AdTGF-β1. While in AdTGF-β1 collapse of septal walls was linked with a decrease in open alveolar number per lung (Fig. 7B) the collapse of septa in AdCl resulted in smaller alveoli (Fig. 7C). ΔH (the difference between H_end_ and the first tissue elastance value after recruitment) trended to higher values with decreasing PEEP ventilation, in particular in AdTGF-β1. In the latter group, the mean thickness of interalveolar septa was found to correlate positively (r = 0.73) and the number of open alveoli negatively (r = − 0.49 p = 0.15) with ΔH at low PEEP levels. The increase in H_end_ with elevating PEEP ventilation from 5 to 12 cmH_2_O in AdCl and AdTGF-β1 (Fig. 3D) correlated in both groups negatively with the density, per unit of parenchyma, of alveolar surface area and number: the smaller the surface area or numerical density the higher the corresponding H_end_ (Fig. 8D,E).

Taken together, the increase in H with reducing PEEP from 5 to 0 cmH_2_O is linked to alveolar derecruitment in AdTGF-β1 and the formation of pleats in the septal walls independent of alveolar derecruitment in AdCl. With PEEP levels decreasing towards 0 cmH_2_O, ΔH reflects alveolar instability. Septa of collapsed alveoli fold on each other so that these appeared to be thickened, a finding, which correlated with decrease in the number of open alveoli in AdTGF-β1. The increase in H_end_ with PEEP increasing from 5 to 12 cmH_2_O was inversely correlated to the alveolar surface area density, indicating distension of the parenchyma.

### BAL analysis data

**Figure 9:**
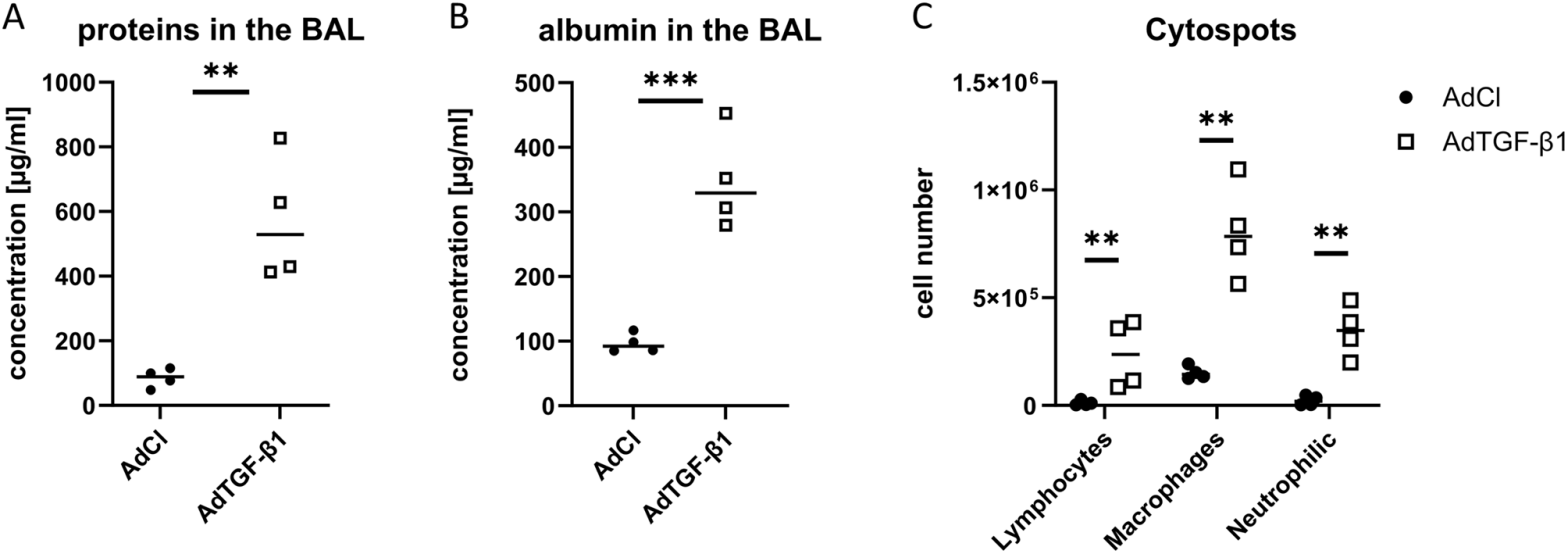
Biochemical analyses of the bronchoalveolar lavage. The proteins (**A**) as well as the albumin (**B**) concentrations show significant increases in the AdTGF-β1 mice compared to AdCl. The numbers of lymphocytes, macrophages/monocytes and neutrophils per BAL increases in the AdTGF-β1 animals compared to AdCl (**C**). (*p ≤ 0.05, **p ≤ 0.01, ***p ≤ 0.001, ****p ≤ 0.0001).

Four animals per group were used for the BAL analysis. As expected, the total protein concentration of the BAL was much higher in AdTGF-β1 than in AdCl animals (Fig. 9A) and BAL albumin content was three to four times higher in AdTGF-β1 than in AdCl animals (Fig. 9B). Because albumin is a marker for the damage of the blood-gas barrier, the results clearly imply a damage of this barrier linked with vascular leakage in the AdTGF-β1 animals. Cytological differentiation of BAL cells show that, in the AdCl group, macrophages comprise the largest fraction. All cell types were increased in the AdTGF-β1 group, but the lymphocytes and neutrophils showed a greater increase so that the fraction of macrophages was lowered in AdTGF-β1 compared to AdCl (Fig. 9C).

## Discussion

The optimal PEEP for mechanical ventilation of injured lungs is a balancing act between different, potentially harmful mechanisms. If PEEP is too low, atelectrauma and stress concentration might propagate lung injury; if PEEP is too high, overdistension of distal airspaces might be critical^2,36^. To understand the link between PEEP and these lung injury pathways the aims of the present study were: 1. to investigate the relationship between alveolar recruitment, parenchymal distension and end-expiratory airway pressure by means of quantitative morphology and 2. to analyze how recruitment and distension are reflected in lung mechanical properties in injured and healthy lungs. For these purposes, lung mechanical function and stereological data were determined at different PEEPs and corresponding airway opening pressures (Pao) and correlated with each other in mice injured with AdTGF-β1 or empty vector (AdCl, control).

Compared to controls, alveolar instability was markedly increased after AdTGF-β1 challenge. With a maximum number of open alveoli determined from the AdCl group at Pao = 20 cmH_2_O a decrease in Pao to 2 cmH_2_O was linked with a reduction in alveolar number by 26% and 76% in AdCl and AdTGF-β1, respectively (Table 2). Note that a maximum alveolar recruitment of 73% was achieved in the AdTGF-β1 group at Pao = 20 cmH_2_O.

The fraction of collapsed septa was also used to analyze alveolar recruitability. After a recruitment maneuver, the fraction of collapsed alveolar septa increased with decreasing Pao on expiration. This was an important contributor to the loss of alveolar volume in both the AdCl and AdTGF-β1 groups (Fig. 7A). However, the underlying mechanisms responsible for the increased fraction of collapsed septa were different between groups. In the AdTGF-β1 group the increased collapse was primarily due to the loss of complete alveoli which is reflected in the correlation between the fraction of collapsed alveolar septa and alveolar number (Fig. 7B). In AdCl, collapsed septa and the number-weighted mean alveolar volume (= mean alveolar size) were correlated (Fig. 7C): septal wall folding resulted in a decrease in alveolar size. However, this correlation was relatively weak, indicating that diverse micromechanical mechanisms are involved in size change of the alveolar compartment in the AdCl group. In addition to partial and complete alveolar collapse, shape changes and stretching of the interalveolar septa are mechanisms of alveolar volume change^37,38^.

Considering the Pao effects in AdCl, differences in relative volume changes were detectable in the alveolar and the alveolar duct airspace compartments. When increasing Pao from 2 to 20 cmH_2_O the volume of the ductal airspace tripled while the alveolar volume doubled (Table 2). Alveolar ducts are bordered by a meshwork of alveolar entrance rings while alveoli are limited by the interalveolar septa. Hence, volume change in the ductal airspaces is primarily achieved by stretching of this meshwork, whereas volume change in alveolar compartment is accommodated by continuous interalveolar septa, including the delicate blood–gas barrier, via shape changes, unfolding of pleats or stretching. These findings are similar to results published previously in healthy mouse and rat lungs^12,39^. In AdTGF-β1 lungs, increasing Pao from 2 to 20 cmH_2_O increased the volume of the alveolar compartment by a factor of 3.6, which is greater than in AdCl, while the alveolar duct compartment behaved quite similarly to AdCl. Unlike to AdCl, the increase in volume of the alveolar compartment in AdTGF-β1 did not coincide with an increase in the number-weighted mean alveolar volume so that it can be concluded that a dominant mechanism for the gain of alveolar airspace is recruitment of alveoli. Furthermore, these alveolar recruitment processes led to a near doubling of the alveolar surface area in AdTGF-β1 lungs, which was a much higher relative increase than observed in AdCl.

During PEEP ventilation in the range of 5–8 cmH_2_O the tissue elastance was minimized in both study groups. At PEEPs higher or lower than 5–8 cmH_2_O the AdTGF-β1 and to a lesser extent the AdCl lungs showed increased elastance (Fig. 3D). At lower lung volumes, e.g. with PEEP < 5 cmH_2_O, folding/ derecruitment of septal walls was linked with higher tissue elastance in both groups (Fig. 8B,C). In AdTGF-β1, increased H was associated with alveolar derecruitment as indicated by the number of open alveoli. This observation aligns with previous studies in bleomycin-induced or ventilation-induced lung injury^11,12,39,40^. In the AdCl group the increased elastance at low PEEP was associated with an increased fraction of collapsed septal tissue but not a reduction in the number of open alveoli. This suggests that in AdCl alveoli only partially derecruited so that the alveolar entrance remained open and identifiable for stereological counting. The formation of these septal pleats predominantly occurs at the junctions of interalveolar septa and has been suggested to be a physiological mechanism of the alveoli to adapt to volume changes without stretching the vulnerable blood-gas barrier^38^.

The quasi-static pressure–volume relationship on expiration demonstrates a strong nonlinearity at airway opening pressures of about 10 cmH_2_O (Fig. 3H), a phenomenon usually explained by the network of collagen fibers which becomes progressively stress bearing in recruited septa with higher lung volumes^37^. Stretch of the interalveolar septa causes stiffening that limits increases in alveolar volume and surface area. As a result of stiffening and a volume shift to the ducts, the septal surface area density (the surface area per unit volume of parenchyma) declined in AdTGF-β1 and AdCl with increasing Pao. Due to the failure of unfolding of septal walls and a reduced amount of recruited septa, the lowest alveolar surface area densities were found in AdTGF-β1 at Pao 10 and 20 cmH_2_O. The jump in tissue elastance with increasing PEEP from 5 to 12 cmH_2_O correlated with the decline in alveolar surface area density at the corresponding Pao in both AdTGF-β1 and AdCl (Fig. 8D,E). This indicates that at the upper part of the pressure–volume relationship, the reduction in alveolar surface density reflects mechanical tensioning of lung parenchyma, a mechanism which was much more pronounced in AdTGF-β1 than in AdCl.

The decrease in alveolar surface density is considered as a surrogate marker for progressive airspace dilatation. The distribution of chord lengths support this assertion and shows a right shift in the AdTGF-β1 group at Pao below 10 cmH_2_O, suggesting some septal overextension in the presence of widespread microatelectases which may be explained by alveolar interdependence^41^. Also, due to the AdTGF-β1 induced downregulation of hydrophilic surfactant proteins, surface tension increases and destabilizes predominantly smaller alveoli so that larger alveoli remain open. This reasoning can also explain the right shifted distribution of chord length at Pao < 10 cmH_2_O in AdTGF-β1 compared to AdCl.

With increasing Pao or PEEP, distal airspaces are recruited so that with a Pao of 10 cmH_2_O the chord length distributions are nearly identical in AdTGF-β1 and AdCl. However, chord lengths include both ductal and alveolar airspaces, and it is impossible to determine whether there is compartment-specific and even regionally increased distension. Since the alveoli and ducts have distinct morphological boundaries, and thus micromechanical properties, a differential analysis is desirable. While the ductal volume did not differ between the AdCl and AdTGF-β1 groups, the alveolar compartment showed significant differences. Volumetric strain at the alveolar level with increasing Pao is reflected by increase in the number-weighted mean alveolar size. In the AdCl group a progressive increase from Pao = 2 to 20 cmH_2_O resulted in an increase in number-weighted alveolar volume from 68,500 to 106,500 µm^3^, which corresponded to a volumetric and linear strain of roughly 55% and 15%, respectively. These values are within the range found in the literature for healthy lungs^42^. In the AdTGF-β1 group, however, the mean alveolar size was virtually independent of Pao.

With progressive increase in Pao, alveolar recruitment, shape change and stretching are likely to occur in parallel. Even though alveoli were successfully recruited with Pao up to 20 cmH_2_O in AdTGF-β1, the fraction of collapsed septa remained comparably high (Fig. 5E). It is plausible that alveoli recruit merely in part, e.g. by emerging in a way that the alveolar entrance rings are open to the alveolar duct parts of the alveolus remain derecruited. Hence, partly recruited alveoli co-exist with those that are completely open and at risk to being overstretched with larger PEEP. These alterations are not reflected in the chord length measurements since this parameter does not distinguish between alveolar ducts and alveolar airspaces^35^.

In AdTGF-β1 the increase in tissue elastance at PEEP < 5 cmH_2_O is due, at least in part, to alveolar instability as indicated by a decrease in the number of open alveoli and an increase in septal wall thickness. At higher PEEPs the increase in tissue elastance is linked with airspace distension as reflected by a decrease in alveolar surface area density. Hence, the optimal PEEP for mechanical ventilation of AdTGF-β1 appears to be in the range of 5–8 cmH_2_O. Morphological data agues for an optimal PEEP of 8 rather than 5 cmH_2_O since at PEEP = 5 cmH_2_O there is increased heterogeneity in acinar airspaces, and thus evidence for heterogenous ventilation. Moreover, there is still potential for alveolar recruitment with PEEP larger than 5 cmH_2_O.

Previous clinical studies used PEEP titration and pulmonary imaging to investigate the recruitability of collapsed lung parenchyma as well as the degree of overdistension in ARDS patients with the goal to discern the optimal PEEP to mitigate VILI^15,43,44^. Electrical impedance tomography (EIT) can be used to monitor regional aeration and to estimate local (= voxelwise) dynamic compliance making this a functional, rather than an anatomical approach^45^. Starting from the PEEP level at which local dynamic compliance is highest, a decrease in dynamic compliance with lower and larger PEEP levels is interpreted as a derecruitment and overdistension of acinar airspaces, respectively^14^. To identify an optimal ventilation PEEP, Costa et al*.* balanced parenchymatous recruitment against overdistension and chose the crossing point of both processes as optimal PEEP^14^. In the present study, we applied a similar approach based on organ-scale tissue elastance and stereological data. Using high resolution computed tomography (HRCT) for imaging of ARDS patients at different PEEP levels and quantification of the amount of recruitable lung parenchyma, up to 45% could be re-aerated in individual patients with appropriate PEEP levels^16,17^. Accordingly, with a potential to recruit 48% of collapsed alveoli, the AdTGF-β1 lungs could be considered as high recruiters reflecting the situation in a subgroup of ARDS patients who have a high potential of parenchymal recruitability with increasing PEEP.

Basically, VILI results in injury of the blood-gas barrier so that its micromechanical features are of relevance. These can be described as unfolding/folding, stretching/de-stretching and shape changes independent from the other two mechanisms. In presence of high surface tension, unfolding processes cause potentially harmful fluid-mechanical forces operating on the delicate blood–gas barrier^5,46^. This mechanism has to be distinguished from real stretching, the mechanism of volutrauma, which results in an increase of the surface area of the basal membrane which forms a carpet underneath the alveolar epithelium. To discriminate these two mechanism from each other, electron microscopic quantification of the surface area of the epithelial basal lamina at different Pao would be necessary^38^. Hence, it is difficult to deduce from light microscopic investigation whether or not the reduced surface density of alveoli with higher Pao is really linked to relevant/injurious stretch of the blood-gas barrier as indicated by the lung mechanical properties showing stiffening of lung parenchyma.

In conclusion, AdTGF-β1 increased alveolar instability as shown by loss of open alveoli with decreasing pressures. However, approximately 48% of collapsed alveoli were recruitable with end-expiratory pressures as high as 20 cmH_2_O. Hence, this animal model is characterized by high alveolar recruitability. Minimal tissue elastance was reached with PEEP 5–8 cmH_2_O in AdTGF-β1 and AdCl, a finding which reproduces previous studies and suggests optimal tissue configuration also in the healthy lung^47^. Both reduction and increase in PEEP resulted in higher tissue elastance. At low PEEPs this stiffening was due to progressive loss of alveolar air by septal wall folding or alveolar collapse. At high PEEPs the increase is attributed to stiffening of septal walls indicated by a failure to increase alveolar surface area proportionally to volume. Considering lung mechanical and stereological data, a least injurious mechanical ventilation featured by a balance of recruitment and distension might be achieved with PEEP = 8 cmH_2_O.
